# Understanding psychological help-seeking attitudes among individuals with sports injuries: the role of psychological risk and protective factors

**DOI:** 10.3389/fpsyg.2026.1856848

**Published:** 2026-06-02

**Authors:** Serhat Erail, Mustafa Selim Altınışık

**Affiliations:** 1 Department of Sports Management, Faculty of Sports Sciences, Sports Management Division, Ondokuz Mayis University, Atakum, Samsun, Türkiye; 2 Department of Educational Sciences, Faculty of Education, Division of Psychological Counseling and Guidance, Ondokuz Mayis University, Atakum, Samsun, Türkiye

**Keywords:** athlete burnout, attitudes toward seeking psychological help, injury anxiety, mediation analysis, psychological recovery

## Abstract

**Introduction:**

This study aimed to examine psychological help-seeking attitudes among individuals who had experienced sports injuries within the framework of psychological risk factors and protective psychological resources.

**Methods:**

A total of 409 participants with a history of sports injury were included in the study. Data were collected using measures of attitudes toward seeking professional psychological help, athlete burnout, psychological recovery in sport, mental health continuity, coping strategies, and injury anxiety. Hierarchical multiple regression and mediation analyses were conducted.

**Results:**

The findings showed that injury anxiety, coping strategies, psychological recovery, and mental health continuity positively predicted psychological help-seeking attitudes, whereas athlete burnout negatively predicted these attitudes. The mediation analysis further revealed that athlete burnout played a suppressing mediating role in the relationship between injury anxiety and psychological help-seeking attitudes. Specifically, injury anxiety directly increased psychological help-seeking attitudes but indirectly weakened them through athlete burnout.

**Discussion:**

Overall, the results suggest that psychological help-seeking after sports injury is not shaped solely by psychological distress, but by a multidimensional process involving psychological resources and burnout.

## 1 Introduction

Sports injuries are a common experience across various disciplines and competitive levels, often leading to significant disruptions in individuals' lives (Timpka et al., 2014). In this context, attention is typically directed toward the medical and physical dimensions of injury. Indeed, a substantial body of research has primarily focused on aspects such as the nature of physical damage, performance-related processes, injury prevention, and physical rehabilitation (Stephenson et al., 2021; O'Brien et al., 2019; Verhagen and Van Mechelen, 2010; Caine et al., 2008; Timpka et al., 2006; Chard and Lachmann, 1987). However, although sports injuries are generally conceptualized as physical conditions, they may also lead to significant psychological consequences for athletes (Heil, 1993). Following injury, athletes often encounter numerous changes characterized by uncertainty, which may substantially alter their daily functioning (Podlog et al., 2013).

The experience of injury can disrupt daily routines and temporarily suspend athletes' sense of athletic identity (Brewer and Chatterton, 2024). Research has further shown that injury may influence individuals' interests and reduce engagement in previously enjoyable activities (Malcolm and Pullen, 2020). Such changes can significantly affect athletes' emotional states and overall psychological wellbeing (Jeong and Li, 2024; Haugen, 2022). In addition, accompanying physical pain may undermine psychological resilience (Mojtahe and Burrichter, 2023; Ernst et al., 2022; Levy et al., 2006). Collectively, these challenges may lead to diminished self-confidence, self-esteem issues, depressive symptoms, heightened anxiety, loss of motivation, frustration, and emotional instability (Dzieciatkowska et al., 2025; Kaplánová, 2024; Bani-Irshid and Bani-Rshaid, 2022; Podlog and Eklund, 2005). Moreover, injury experiences may increase the risk of trauma-related responses characterized by re-experiencing, avoidance of sport-related activities, and hyperarousal, which are consistent with post-traumatic stress dynamics (Aron et al., 2019; Bateman and Morgan, 2019; Padaki et al., 2018; Bailes and Hudson, 2001). In particular, fear of re-injury and performance-related concerns may psychologically complicate the return-to-sport process (Covassin et al., 2015; Walker et al., 2010).

These psychological responses following injury may not be experienced uniformly by all athletes. Some individuals are able to manage the recovery process more adaptively through effective coping strategies. Research has demonstrated that processes such as post-traumatic growth and social support can facilitate psychological adjustment following injury (Vann et al., 2018; Green and Weinberg, 2001). However, for some athletes, the injury experience may also become a significant source of psychological stress that can adversely affect performance (Nippert and Smith, 2008). Overall, considering the potential impact of injury on mental health, the recovery process can be psychologically challenging and difficult to manage. This highlights the importance of psychological support following sports injuries. Indeed, receiving psychological support at an early stage after distressing life events, such as injury, has been shown to enhance individuals' functioning and mental health (McNally et al., 2003). Nevertheless, help-seeking behaviors are influenced by a range of economic, cultural, social, and individual factors.

Psychological help-seeking intention is considered one of the most important antecedents of actual help-seeking behavior (Topkaya, 2014). Although the literature on athletes' help-seeking intentions following injury is limited, it is suggested that this process may be influenced by various individual and social factors (Putukian, 2016). One of the most salient of these factors may be athlete burnout. Prolonged exposure to physical and psychological demands may deplete athletes' emotional resources, thereby reducing their likelihood of seeking help (Gustafsson et al., 2017; Li et al., 2013). In addition, fear of re-injury may lead athletes to exhibit avoidance behaviors, which can hinder their psychological coping processes by promoting disengagement from sport-related cues (Turgut et al., 2025; Kvist et al., 2005). In some cases, beliefs that difficulties are insurmountable may even lead athletes to withdraw from sport entirely (Ristolainen et al., 2012).

In this context, coping strategies play a crucial role in regulating psychological responses following injury. Functional coping strategies have been shown to positively influence psychological help-seeking intentions (Niegocki and Ægisdóttir, 2019; Panis et al., 2019). Moreover, research indicates that coping strategies are closely associated with psychological recovery and wellbeing among athletes (Osborne and Doty, 2022; Masten et al., 2014; Green and Weinberg, 2001). However, given that athletes generally demonstrate relatively low levels of willingness to seek psychological help, examining help-seeking intentions in the context of sports injuries remains particularly important (Jones et al., 2022; Putukian, 2016).

A review of the literature indicates that numerous studies have examined the psychological dimensions of sports injuries. However, findings regarding the factors influencing psychological help-seeking intentions among injured athletes remain limited. Existing research has predominantly focused on variables such as injury-related responses, gender, and expectancy beliefs, highlighting the need for more comprehensive investigations (Putukian, 2016; Hoar and Flint, 2008; Stadden, 2007).

In contrast, psychological help-seeking intentions during the injury process are likely shaped by the simultaneous interaction of multiple psychological dynamics. Therefore, adopting a more integrative approach that considers these variables collectively may provide a more comprehensive understanding and contribute significantly to the literature. In this regard, examining psychological help-seeking attitudes among individuals who have experienced sports injuries within a multidimensional framework represents an important research need and aims to address a critical gap in the field.

Given the central role of injury-related anxiety in athletes' psychological responses to injury, the present study specifically examined athlete burnout as a potential mediator in the relationship between injury anxiety and psychological help-seeking attitudes. Injury anxiety may increase athletes' awareness of vulnerability, uncertainty, and the need for professional psychological support. However, prolonged injury-related anxiety may also contribute to athlete burnout by intensifying emotional exhaustion, reduced motivation, and psychological withdrawal. In turn, burnout may weaken athletes' willingness or readiness to seek help. Therefore, examining athlete burnout as a mediator between injury anxiety and psychological help-seeking attitudes provides a theoretically meaningful way to understand the dual role of injury anxiety in the help-seeking process.

Accordingly, the following hypotheses were proposed:

H1: Education level, income level, and knowledge about psychological help significantly differentiate attitudes toward seeking psychological help.
H2: Injury anxiety positively and significantly predicts attitudes toward seeking psychological help.
H3: Athlete burnout significantly predicts attitudes toward seeking psychological help.
H4: Mental health continuity in athletes positively and significantly predicts attitudes toward seeking psychological help.
H5: Coping strategies positively and significantly predict attitudes toward seeking psychological help.
H6: Psychological recovery in sport positively and significantly predicts attitudes toward seeking psychological help.
H7: Athlete burnout mediates the relationship between injury anxiety and attitudes toward seeking psychological help.

## 2 Method

### 2.1 Study Design

This study was designed using a quantitative research approach to examine psychological help-seeking intentions among athletes who have experienced sports injuries. The study was based on a correlational survey model, aiming to investigate the variables associated with psychological help-seeking intentions.

In this context, coping strategies, psychological recovery in sport, athlete burnout, and injury anxiety were identified as independent variables, while psychological help-seeking attitudes were considered the dependent variable. In addition, the mediating role of athlete burnout in the relationship between injury anxiety and psychological help-seeking attitudes was examined.

Accordingly, both multiple linear regression and mediation analyses were conducted. The proposed model aimed to test both the direct and indirect relationships among the study variables.

### 2.2 Participants

The study sample consisted of athletes aged between 18 and 55 years (*M* = 23.35) who had previously experienced a sports injury or were currently undergoing an injury process. The sample was determined using purposive sampling. Accordingly, the inclusion criteria required participants to be actively engaged in sports and to have experienced at least one sports injury.

Purposive sampling was preferred because it allows access to individuals with characteristics directly relevant to the research objectives. The final sample included 409 participants, comprising 189 females and 220 males. Detailed demographic characteristics of the participants are presented in Table 1.

**Table 1:** Demographic characteristics of the participants.

Variable		*n*	%
Education level	High school graduate	249	60.8
	Undergraduate degree	127	31.1
	Graduate degree	33	8.1
Income level	Low	49	12.0
	Medium	298	72.9
	High	62	15.2
Type of sport	Individual sports	219	53.5
	Team sports	190	46.5
Years of sport participation	0–3 years	65	15.9
	4–6 years	85	20.8
	7–9 years	101	24.7
	10 years and above	158	38.6
Level of participation	Recreational	60	14.7
	Amateur	172	42.1
	Semi-professional	85	20.8
	Professional	92	22.5
Number of injuries	1	156	38.1
	2	93	22.7
	3	81	19.8
	4 and above	79	19.3
Time away after injury	Less than 1 month	176	43.0
	1–6 months	137	33.5
	7 months or more	96	23.5
Knowledge of psychological counseling	Low	136	33.3
	Medium	229	56.0
	High	44	10.8
Previous psychological counseling experience	Yes	125	30.6
	No	284	69.4

*N* = 409.

### 2.3 Measures

***Demographic Information Form***. A demographic information form was developed by the researchers to collect data that would facilitate the examination of psychological help-seeking attitudes among individuals who have experienced sports injuries. The form included items related to participants' age, gender, education level, income level, type and level of sport participation, sport history, injury experiences, and prior experience with psychological help.

***Attitudes Toward Seeking Professional Psychological Help Scale—Short Form*** ***(ATSPPH-SF)***. The Attitudes Toward Seeking Professional Psychological Help Scale was originally developed by Fischer and Turner (1970) as a 29-item instrument to assess attitudes toward seeking psychological help. It was later shortened by Fischer and Farina (1995) and has been widely used to assess help-seeking tendencies across different samples. The Turkish adaptation was conducted by Topkaya (2010), resulting in a 10-item single-factor structure. The scale is rated on a 4-point Likert scale (0 = strongly disagree, 3 = strongly agree), with total scores ranging from 0 to 27. Higher scores indicate more positive attitudes toward seeking psychological help. The internal consistency coefficient was reported as.84 in the original study and.69 in the Turkish adaptation, while it was calculated as.78 in the present study.

***Athlete Burnout Questionnaire (ABQ)***. The Athlete Burnout Questionnaire was developed by Raedeke and Smith (2001) to assess burnout levels among athletes. The original scale consists of three subdimensions: reduced sense of accomplishment, emotional/physical exhaustion, and devaluation. The Turkish adaptation was conducted by Kelecek et al. (2016), resulting in a 13-item version rated on a 5-point Likert scale (1 = almost never, 5 = almost always). The Turkish version preserves the three-dimensional structure of the original scale. However, in the present study, the total score was used to represent the overall level of athlete burnout. Higher scores indicate higher levels of burnout. The internal consistency coefficient was reported as.87 in the original study and was calculated as.88 in the present study.

***Mental Health Continuum – Short Form (MHC-SF)***. The Mental Health Continuum – Short Form was adapted for athletes by Foster and ve Chow (2019) to assess positive mental health and subjective wellbeing in sport contexts. The Turkish adaptation was conducted by Tingaz (2022). The scale consists of 14 items across three dimensions: emotional wellbeing, psychological wellbeing in sport, and social wellbeing in sport. It is rated on a 6-point Likert scale (0 = never, 5 = every day). Higher scores indicate higher levels of mental health continuity. The internal consistency coefficient was reported as.92 in the adaptation study and.95 in the present study.

***Brief COPE Inventory (B-COPE)***. The Brief COPE Inventory was originally developed by Carver et al. (1989) as a 60-item measure with 15 subdimensions. The Turkish adaptation by Dicle and Ersanli (2015) resulted in a 32-item scale with five factors: self-help, approach, adaptation, avoidance, and self-punishment. The scale is rated on a 4-point Likert scale (1 = I never do this, 4 = I usually do this). Total scores range from 32 to 128, with higher scores indicating stronger coping tendencies. The internal consistency coefficient was reported as.76 in the adaptation study and.86 in the present study.

***Psychological Recovery in Sport Scale (PRS)***. The Psychological Recovery in Sport Scale was developed by Kaygusuz and Karagözoglu (2023) to assess athletes' psychological recovery in response to sport-related demands. The scale consists of 20 items across four dimensions: mental recovery, vitality and energy, psychological detachment, and recovery outcomes. It is rated on a 10-point Likert scale (1 = not at all appropriate, 10 = completely appropriate). Higher scores indicate higher levels of psychological recovery. The internal consistency coefficient was reported as.89 in both the original and the present study.

***Sport Injury Anxiety Scale – Short Form (SIAS-SF)***. The Sport Injury Anxiety Scale was developed by Rex and Metzler (2016) to assess athletes' anxiety related to the possibility of injury. The Turkish adaptation was conducted by Caz et al. (2019). The scale consists of 19 items across six dimensions: fear of losing ability, fear of being perceived as weak, fear of pain, fear of disappointment, fear of losing social support, and fear of re-injury. It is rated on a 5-point Likert scale (1 = strongly disagree, 5 = strongly agree), with total scores ranging from 19 to 95. Higher scores indicate higher levels of injury-related anxiety. The internal consistency coefficient was reported as.88 in the adaptation study and.84 in the present study.

### 2.4 Procedure

First, a comprehensive literature review was conducted to define the research problem and identify the study variables and measurement instruments. Inclusion criteria were established, and permission to use the selected scales was obtained from the original developers or adaptors. Subsequently, ethical approval was obtained from the Ondokuz Mayis University Social and Human Sciences Research Ethics Committee (Approval No: 2025-1484, Date: 28.11.2025).

Following ethical approval, the data collection process was initiated. Participants were informed about the purpose of the study, and informed consent was obtained. Data were collected using both face-to-face and online methods (Google Forms). The data collection process took approximately 20 min per participant. Participants' information was anonymized through coding procedures, and strict confidentiality was maintained throughout the study. All procedures were conducted in accordance with ethical principles, and participation was voluntary.

### 2.5 Data Analysis

The data were analyzed using IBM SPSS Statistics software. Initially, the dataset was examined for missing values and outliers. Participants with excessive missing data or not meeting the inclusion criteria were excluded from the dataset (n = 10). Reverse-coded items were recoded prior to analysis.

Normality assumptions were evaluated based on skewness and kurtosis values, and the data were found to exhibit a normal distribution. Outliers were further assessed using z-scores. The reliability of the scales was evaluated using Cronbach's alpha coefficients, and all scales demonstrated acceptable internal consistency (α ≥.70). Relationships between variables were examined using Pearson correlation analysis.

To determine whether psychological variables predicted psychological help-seeking attitudes after controlling for relevant demographic and background variables, hierarchical multiple regression analysis was conducted. In the first step, age, gender, education level, income level, psychological help knowledge level, and previous psychological counseling experience were entered as control variables. In the second step, athlete burnout, mental health continuity, psychological recovery in sport, injury anxiety, and coping strategies were added to the model. Regression assumptions were assessed using tolerance and VIF values, and no multicollinearity issues were detected.

In addition, mediation analysis was performed using the PROCESS macro developed by Hayes (2017). Model 4 was employed, and the significance of indirect effects was tested using 5,000 bootstrap samples with 95% confidence intervals. The level of statistical significance was set at.05.

## 3 Results

This section presents the findings of the study in line with the research questions and hypotheses. First, the effects of demographic variables on psychological help-seeking attitudes were examined. Subsequently, descriptive statistics and correlations among the study variables were analyzed. Finally, hierarchical multiple regression analysis was conducted to examine whether the psychological variables predicted psychological help-seeking attitudes after controlling for relevant demographic and background variables, and the mediating role of athlete burnout in the relationship between injury anxiety and psychological help-seeking attitudes was examined.

### 3.1 Demographic variables results

First, a Pearson correlation analysis was conducted to examine the relationship between age and psychological help-seeking attitudes, and the results are presented in Table 2.

**Table 2:** Correlation between age and psychological help-seeking attitudes.

Variable	Age	ATSPPH
Age	—	0.208^**^
ATSPPH		—

*N* = 409. ^*^p < .05, ^**^p < .01.

As shown in Table 2, a positive and statistically significant relationship was found between age and psychological help-seeking attitudes (*r* = 0.208, *p* < 0.001). This finding indicates that psychological help-seeking attitudes tend to increase as age increases. However, the strength of the relationship was low, suggesting that age has a limited but significant association with psychological help-seeking attitudes.

A one-way analysis of variance (ANOVA) was conducted to examine whether psychological help-seeking attitudes differ across education levels. The results are presented in Table 3.

**Table 3:** Psychological help-seeking attitudes by education level.

Education level			*n*	*M*	SD
High school graduate			249	26.94	4.65
Bachelor's degree			127	27.73	5.26
Graduate degree			33	29.67	4.90
Source	SS	df	MS	*F*	*p*
Between groups	236.299	2	118.149	4.991	0.007
Within groups	9610.327	406	23.671		
Total	9846.626	408			

Homogeneity of variances was met (Levene's test = 1.226, *p* =.295). Therefore, Tukey *post hoc* test was used.

The results of the one-way ANOVA indicated that psychological help-seeking attitudes differed significantly across education levels, F(2, 406) = 4.991, p =.007. Tukey's *post hoc* analysis revealed that participants with a graduate degree had significantly higher psychological help-seeking attitudes than high school graduates (p < .05). However, no significant differences were found between high school graduates and participants with a bachelor's degree or between participants with bachelor's and graduate degrees (p >.05). Overall, these findings suggest that psychological help-seeking attitudes tend to become more positive as education level increases.

A one-way analysis of variance (ANOVA) was conducted to examine whether psychological help-seeking attitudes differ across income levels. The results are presented in Table 4.

**Table 4:** Psychological help-seeking attitudes by income level.

Income level			*n*	*M*	SD
Low			49	25.84	4.62
Medium			298	27.78	4.95
High			62	26.87	4.74
Source	SS	df	MS	*F*	*p*
Between Groups	179.028	2	89.514	3.759	0.024
Within Groups	9667.598	406	23.812		
Total	9846.626	408			

Homogeneity of variances was met (Levene's test = 0.593, *p* = 0.553). Therefore, Tukey's *post hoc* test was used.

The results of the one-way ANOVA indicated that psychological help-seeking attitudes differed significantly across income levels, [(*F*_(2, 406)_ = 3.759, *p* = 0.024)]. Tukey's *post hoc* analysis revealed that participants with a medium income level had significantly higher psychological help-seeking attitudes than those with a low income level (*p* < 0.05). However, no significant differences were found between low- and high-income groups or between medium– and high-income groups (*p* >0.05).

In addition, a one-way analysis of variance (ANOVA) was conducted to examine whether psychological help-seeking attitudes differ according to participants' level of knowledge about psychological help. The results are presented in Table 5.

**Table 5:** Psychological help-seeking attitudes by psychological help knowledge level.

Knowledge level			*n*	*M*	SD
Low			136	25.90	4.51
Medium			229	28.14	4.82
High			44	28.25	5.57
Source	SS	df	MS	*F*	*p*
Between groups	459.815	2	229.908	9.944	< 0.001
Within Groups	9386.811	406	23.120		
Total	9846.626	408			

Homogeneity of variances was met (Levene's test = 2.105, *p* = 0.123). Therefore, Tukey's *post hoc* test was used.

The results of the one-way ANOVA indicated that psychological help-seeking attitudes differed significantly according to participants' level of knowledge about psychological help, [(*F*_(2, 406)_ = 9.944, *p* < 0.001)]. Tukey's *post hoc* analysis revealed that participants with medium and high levels of knowledge about psychological help had significantly higher psychological help-seeking attitudes than those with low levels of knowledge (*p* < 0.05). However, no significant difference was found between the medium and high knowledge groups (*p* >0.05). These findings suggest that greater knowledge about psychological help is associated with more positive attitudes toward seeking psychological help.

Additional analyses were conducted to examine whether psychological help-seeking attitudes differed according to other demographic, sport-related, and injury-related variables that were not included in the main hypotheses. Independent samples *t*-test results indicated that psychological help-seeking attitudes significantly differed by gender, t (407) = 7.103, *p* < 0.001. Female participants reported higher psychological help-seeking attitudes (*M* = 29.16, SD = 4.61) than male participants (*M* = 25.90, SD = 4.67). In addition, psychological help-seeking attitudes significantly differed according to previous psychological counseling experience, t (407) = 4.011, *p* < 0.001. Participants who had previous psychological counseling experience reported higher psychological help-seeking attitudes (*M* = 28.85, SD = 5.03) than those without such experience (*M* = 26.77, SD = 4.73).

However, psychological help-seeking attitudes did not significantly differ according to type of sport, t(407) = −0.683, p =.495; years of sport participation, [*F*_(3, 405)_ = 1.036, *p* = 0.377]; level of participation, [*F*_(3, 405)_ = 2.458, *p* = 0.062]; number of injuries, [*F*_(3, 405)_ = 0.183, *p* = 0.908]; or time away after injury, [*F*_(2, 406)_ = 0.322, *p* = 0.725]. These findings indicate that, in the present sample, psychological help-seeking attitudes were not significantly differentiated by sport- or injury-related characteristics, including the duration of time away after injury.

### 3.2 Descriptive statistics of the study variables

Descriptive statistics for the study variables are presented in Table 6. The mean score for psychological help-seeking attitudes was 27.40 (SD = 4.91). The mean scores for athlete burnout, mental health continuity, psychological recovery in sport, injury anxiety, and coping strategies were 30.25 (SD = 9.72), 64.51 (SD = 14.42), 128.03 (SD = 28.21), 50.51 (SD = 12.31), and 89.43 (SD = 12.61), respectively. These descriptive findings provide an overview of the distribution of the main study variables before examining the relationships among them.

**Table 6:** Descriptive statistics of the study variables.

Variable	*n*	*M*	SD	Var.	Min.	Max.
ATSPPH	409	27.40	4.91	24.13	13.00	40.00
ABQ	409	30.25	9.72	94.59	13.00	59.00
MHC	409	64.51	14.42	208.17	16.00	84.00
PRS	409	128.03	28.21	796.16	43.00	195.00
SIAS	409	50.51	12.31	151.74	19.00	95.00
B-COPE	409	89.43	12.61	159.15	51.00	128.00

Pearson correlation analysis was conducted to examine the relationships among the study variables, and the results are presented in Table 7.

**Table 7:** Correlations among variables.

Variable	ATSPPH	ABQ	MHC	PRS	SIAS	B-COPE
ATSPPH	—					
ABQ	−0.20^**^	—				
MHC	0.31^**^	−0.53^**^	—			
PRS	0.20^**^	−0.51^**^	0.49^**^	—		
SIAS	0.27^**^	0.30^**^	−0.15^**^	−0.21^**^	—	
B-COPE	0.36^**^	−0.10^*^	0.34^**^	0.08	0.17^**^	—

*N* = 409, ^*^p < 0.05, ^**^p < 0.01.

Pearson correlation analysis was conducted to examine the relationships among the study variables. As shown in Table 7, psychological help-seeking attitudes were negatively and significantly associated with athlete burnout (*r* = −0.20, *p* < 0.01), and positively and significantly associated with mental health continuity (*r* = 0.31, *p* < 0.01), psychological recovery in sport (*r* = 0.20, *p* < 0.01), injury anxiety (*r* = 0.27, *p* < 0.01), and coping strategies (*r* = 0.36, *p* < 0.01).

In addition, athlete burnout was negatively associated with mental health continuity (*r* = −0.53, *p* < 0.01) and psychological recovery in sport (*r* = −0.51, *p* < 0.01), but positively associated with injury anxiety (*r* = 0.30, *p* < 0.01). Mental health continuity was positively associated with psychological recovery in sport (*r* = 0.49, *p* < 0.01) and coping strategies (*r* = 0.34, *p* < 0.01). Overall, the findings indicate that psychological help-seeking attitudes are related not only to psychological risk factors, such as burnout and injury anxiety, but also to protective psychological resources, such as mental health continuity, psychological recovery, and coping strategies.

### 3.3 Hierarchical multiple regression analysis

To examine whether psychological variables predicted psychological help-seeking attitudes after controlling for relevant demographic and background variables, a hierarchical multiple regression analysis was conducted. The results are presented in Table 8.

**Table 8:** Hierarchical regression analysis predicting psychological help-seeking attitudes.

Variable	*B*	SE	β	*t*	*p*
Model 1					
Constant	30.022	1.706	—	17.601	< 0.001
Age	0.119	0.036	0.178	3.355	0.001
Gender	−3.057	0.446	−0.311	−6.862	< 0.001
Education level	0.116	0.405	0.015	0.287	0.774
Income level	0.221	0.428	0.023	0.516	0.606
Psychological help knowledge level	0.773	0.369	0.098	2.094	0.037
Previous psychological counseling exp.	−1.589	0.491	−0.149	−3.237	0.001
Model 2					
Constant	14.970	2.587	—	5.787	< 0.001
Age	0.075	0.031	0.112	2.415	0.016
Gender	−3.104	0.389	−0.315	−7.988	< 0.001
Education level	0.083	0.350	0.011	0.237	0.813
Income level	−0.195	0.374	−0.021	−0.520	0.603
Psychological help knowledge level	0.337	0.326	0.043	1.035	0.301
Previous psychological counseling exp.	−1.168	0.432	−0.110	−2.705	0.007
ABQ	−0.058	0.025	−0.114	−2.291	0.022
MHC	0.051	0.017	0.149	2.913	0.004
PRS	0.023	0.009	0.135	2.760	0.006
SIAS	0.119	0.017	0.298	7.096	< 0.001
B-COPE	0.074	0.017	0.189	4.359	< 0.001

Model 1: *R* = 0.437, *R*^2^ = 0.191, Adjusted *R*^2^ = 0.179, F _(6, 402)_ = 15.853, *p* < 0.001. Model 2: *R* =.636, *R*^2^ =.404, Adjusted *R*^2^ = 0.387, [F_(11, 397)_ = 24.461, *p* < 0.001]. Δ*R*^2^ = 0.213, Δ[*F*_(5, 397)_ = 28.325, *p* < 0.001]. ABQ = Athlete Burnout Questionnaire; MHC = Mental Health Continuity; PRS = Psychological Recovery in Sport; SIAS = Sport Injury Anxiety Scale; B-COPE = Brief COPE Inventory.

To examine whether psychological variables predicted psychological help-seeking attitudes after controlling for relevant demographic and background variables, a hierarchical multiple regression analysis was conducted. In the first step, age, gender, education level, income level, psychological help knowledge level, and previous psychological counseling experience were entered as control variables. In the second step, athlete burnout, mental health continuity, psychological recovery in sport, injury anxiety, and coping strategies were added to the model.

As shown in Table 8, Model 1 was statistically significant, [*F*_(6, 402)_ = 15.853, *p* < 0.001, and explained 19.1% of the variance in psychological help-seeking attitudes (*R*^2^ = 0.191). In this model, age, gender, psychological help knowledge level, and previous psychological counseling experience were significant predictors.

Model 2 was also statistically significant, [*F*_(11, 397)_ = 24.461, *p* < 0.001, and explained 40.4% of the variance in psychological help-seeking attitudes (*R*^2^ = 0.404). The inclusion of psychological variables produced a significant increase in explained variance, Δ*R*^2^ = 0.213, Δ [*F*_(5, 397)_ = 28.325, *p* < 0.001. In the final model, injury anxiety (β = 0.298, *p* < 0.001), coping strategies (β = 0.189, *p* < 0.001), mental health continuity (β = 0.149, *p* = 0.004), and psychological recovery in sport (β = 0.135, *p* = 0.006) positively and significantly predicted psychological help-seeking attitudes. Athlete burnout negatively and significantly predicted psychological help-seeking attitudes (β = –.114, *p* = 0.022). In addition, age, gender, and previous psychological counseling experience remained significant predictors in the final model. No multicollinearity problem was detected, as VIF values were within acceptable limits.

### 3.4 Mediation analysis results

To examine the mediating role of athlete burnout in the relationship between injury anxiety and psychological help-seeking attitudes, a mediation analysis was conducted using Hayes' PROCESS macro, Model 4. The significance of the indirect effect was evaluated using 5,000 bootstrap samples and 95% confidence intervals.

As shown in Table 9, injury anxiety had a positive and significant effect on athlete burnout (*B* = 0.2397, SE = 0.0373, *t* = 6.427, *p* < 0.001). Athlete burnout negatively predicted psychological help-seeking attitudes (*B* = −0.1544, *p* < 0.001). The total effect of injury anxiety on psychological help-seeking attitudes was also positive and significant (*B* = 0.1085, *p* < 0.001). When athlete burnout was included in the model, the direct effect of injury anxiety on psychological help-seeking attitudes remained significant and increased in magnitude (*B* = 0.1455, *p* < 0.001).

**Table 9:** Mediating role of ABQ between SIAS and ATSPPH.

Variable	*B*	SE	*t*	*p*	LLCI	ULCI
Model 1: M (ABQ)						
SIAS → ABQ	0.2397	0.0373	6.427	< 0.001	0.1664	0.3130
Model 2: Y (ATSPPH)						
SIAS → ATSPPH (c')	0.1455	—	—	< 0.001	—	—
ABQ → ATSPPH	−0.1544	—	—	< 0.001	—	—
Total effect (c)						
SIAS → ATSPPH	0.1085	—	—	< 0.001	—	—
Indirect effect			Effect	BootSE	BootLLCI	BootULCI
SIAS → ABQ → ATSPPH			−0.0370	—	−0.0551	−0.0225
Completely standardized indirect effect			Effect	BootSE	BootLLCI	BootULCI
SIAS → ABQ → ATSPPH			−0.0928	0.0205	−0.1370	−0.0561

SIAS = Sport Injury Anxiety Scale; ABQ = Athlete Burnout Questionnaire; ATSPPH = Attitudes Toward Seeking Professional Psychological Help Scale.

Bootstrap analysis indicated that the indirect effect of injury anxiety on psychological help-seeking attitudes through athlete burnout was significant, as the confidence interval did not include zero [*B* = −0.0370, 95% (CI −0.0551, −0.0225)]. The completely standardized indirect effect was also significant [*B* = −0.0928, BootSE = 0.0205, 95% (CI −0.1370, −0.0561)]. These findings indicate that athlete burnout has a suppressing mediating role in the relationship between injury anxiety and psychological help-seeking attitudes. In other words, although injury anxiety directly increases psychological help-seeking attitudes, it indirectly decreases these attitudes by increasing athlete burnout.

**Figure 1:**
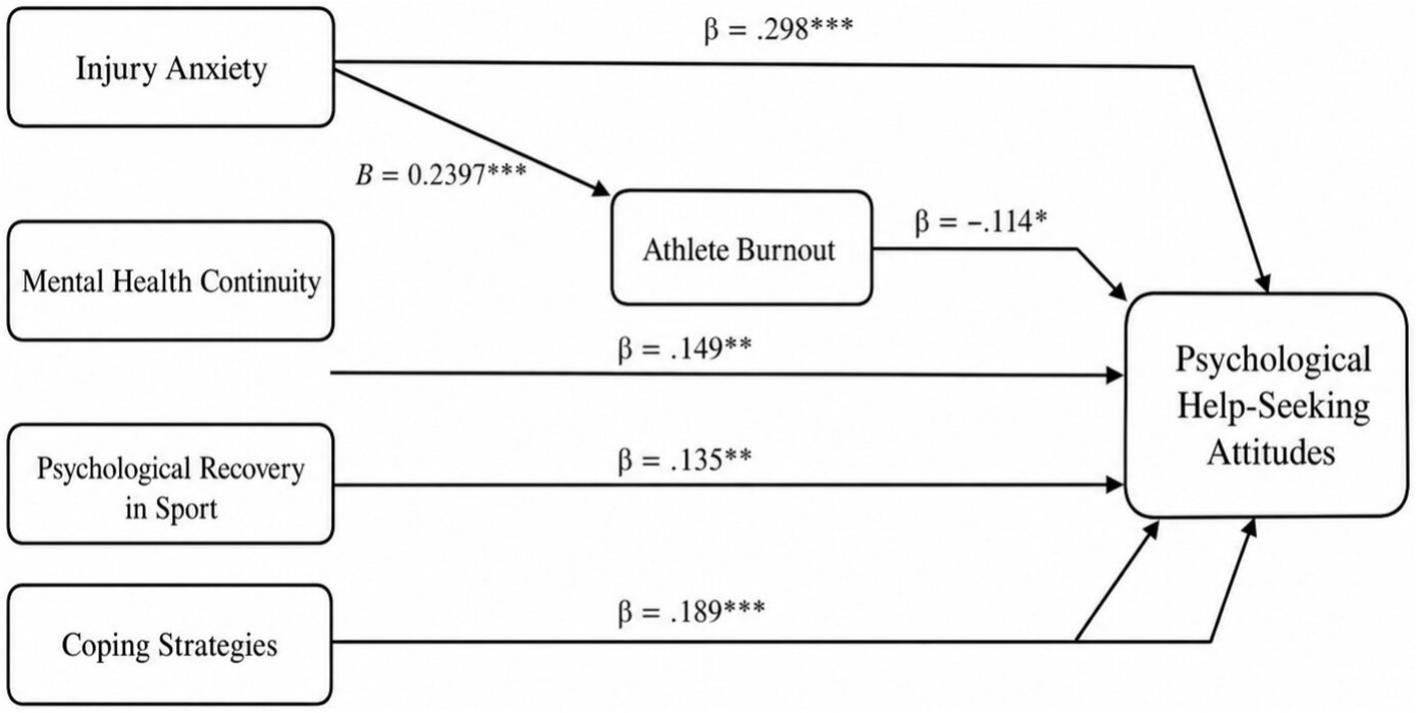
Direct and indirect predictors of psychological help-seeking attitudes. Standardized beta coefficients are presented for the direct paths, whereas the path from injury anxiety to athlete burnout is shown with the unstandardized coefficient. The indirect effect of injury anxiety on psychological help-seeking attitudes through athlete burnout was significant [B = −0.0370, 95% CI (−0.0551, −0.0225)]. *p < 0.05, **p < 0.01, ***p < 0.001.

The direct and indirect relationships among the main study variables are summarized in Figure 1.

## 4 Discussion

The findings of the present study revealed that age, education level, income status, and level of knowledge about psychological help significantly influence psychological help-seeking attitudes. In particular, the results indicated that attitudes toward seeking psychological help become more positive with increasing age. This finding may be explained by the development of greater psychological awareness and a more mature perspective on help-seeking as individuals grow older, consistent with previous research (Mackenzie et al., 2019; Diehl et al., 2014; Nam et al., 2013; Mackenzie et al., 2006).

In addition, higher levels of education and knowledge were associated with more positive attitudes toward psychological help-seeking. This suggests that increased awareness of psychological processes and available support mechanisms facilitates help-seeking behavior (Zhao et al., 2025; Yeap and Low, 2009; Murstein and Fontaine, 1993; Fischer and Cohen, 1972). These findings align with prior studies emphasizing the role of cognitive awareness and education in shaping help-seeking attitudes (Moreno and Moreno, 2024; Picco et al., 2016; Perenc and Radochonski, 2016; Yi and Tidwell, 2005).

Regarding income level, individuals in the middle-income group demonstrated more favorable attitudes compared to those in the low-income group. This pattern may reflect a context in which basic economic needs are sufficiently met while psychological needs become more salient, thereby facilitating help-seeking tendencies (Labra et al., 2019). Overall, these results suggest that psychological help-seeking attitudes are shaped not only by individual needs but also by broader socioeconomic and cognitive factors.

Additional analyses also showed that psychological help-seeking attitudes differed according to gender and previous psychological counseling experience. Female participants and those who had previous psychological counseling experience reported more positive attitudes toward seeking psychological help. This finding suggests that prior familiarity with psychological support may reduce uncertainty and increase openness to future help-seeking. In contrast, no significant differences were found according to sport-related and injury-related variables, including type of sport, years of sport participation, level of participation, number of injuries, and time away after injury. Although time away after injury may theoretically influence psychological responses during recovery, the present findings suggest that it did not significantly differentiate help-seeking attitudes in this sample. This result should be interpreted cautiously, as the cross-sectional design may not fully capture psychological changes across different stages of injury recovery.

Beyond demographic and background variables, the hierarchical regression findings indicate that the psychological variables included in the model explained psychological help-seeking attitudes in a meaningful and multidimensional manner. This finding suggests that injury anxiety, athlete burnout, mental health continuity, psychological recovery in sport, and coping strategies contributed to psychological help-seeking attitudes even after controlling for age, gender, education level, income level, psychological help knowledge level, and previous psychological counseling experience. Notably, the positive effect of injury anxiety suggests that uncertainty following injury, concerns about performance loss, and fear of re-injury may motivate individuals to seek professional support. In this context, anxiety can be considered a risk indicator that triggers help-seeking behavior (Hadad, 2026).

However, the negative effect of athlete burnout highlights that psychological distress does not always lead to increased help-seeking. Burnout reflects not only elevated stress but also diminished motivation, emotional exhaustion, and withdrawal tendencies (Akhrem and Gazdowska, 2016; Eklund and DeFreese, 2015). Consequently, individuals experiencing burnout may disengage from help-seeking despite needing support (Cai et al., 2025). This finding underscores that help-seeking behavior is influenced not only by the severity of problems but also by individuals' psychological energy and motivational capacity.

In contrast, the positive effect of psychological recovery in sport, together with mental health continuity and coping strategies, emphasizes the critical role of psychological resources in facilitating help-seeking. Individuals with higher levels of mental health continuity may possess greater psychological awareness, enabling them to recognize their needs earlier and seek appropriate support (Machado and Bandeira, 2015; Keyes, 2002). Similarly, psychological recovery capacity and adaptive coping strategies may allow individuals to perceive professional support not as a sign of weakness but as an effective means of managing challenging experiences (Lien et al., 2024; Yang et al., 2023; Jones et al., 2020; Griffiths et al., 2011). In this sense, psychological recovery does not appear to reduce the tendency to seek help; rather, it may strengthen individuals' readiness to use psychological support as an adaptive resource.

Taken together, these findings suggest that psychological help-seeking attitudes are not solely determined by the level of distress experienced by individuals, but are also shaped by their psychological resources, coping capacity, and motivational state (Kenny et al., 2016). Accordingly, help-seeking attitudes can be conceptualized as a multidimensional process emerging from the interaction between risk factors and protective psychological resources (Mackenzie et al., 2014; Surgenor, 1985).

The mediation analysis further demonstrated that athlete burnout played a suppressing mediating role in the relationship between injury anxiety and psychological help-seeking attitudes. This finding indicates a dual psychological mechanism. On the one hand, injury anxiety may directly increase psychological help-seeking attitudes by making athletes more aware of vulnerability, uncertainty, performance-related concerns, and the need for professional support. On the other hand, when injury anxiety is accompanied by athlete burnout, this help-seeking tendency may be weakened through emotional exhaustion, reduced motivation, and psychological withdrawal. Therefore, injury anxiety appears to function both as a signal of need and, indirectly through burnout, as a pathway that may undermine readiness to seek help.

This paradox can be explained by the motivational and emotional characteristics of burnout. Previous research has shown that injury-related anxiety increases uncertainty, concerns about performance decline, and fear of re-injury among athletes (Rogers et al., 2024; Chase et al., 2005; Hardy, 1992). These concerns may increase the perceived need for professional psychological support. However, if such anxiety becomes chronic and is accompanied by burnout, athletes may experience emotional depletion, reduced sense of accomplishment, and devaluation of sport participation (Yang et al., 2024; Gustafsson et al., 2017; Eklund and DeFreese, 2015; Raedeke and Smith, 2001). In this state, athletes may recognize their distress but lack the psychological energy, hope, or motivation required to initiate help-seeking. Thus, burnout may suppress the otherwise positive relationship between injury anxiety and help-seeking attitudes.

In this respect, athlete burnout can be conceptualized as an inhibiting psychological mechanism in the help-seeking process (Edwards and Crisp, 2017). It may not eliminate the need for support; rather, it may reduce the individual's capacity to act on that need. This interpretation is consistent with research suggesting that burnout is associated with emotional exhaustion, disengagement, and reduced help-seeking tendencies (Dyrbye et al., 2021; Koutsimani et al., 2019; Gomes et al., 2017). In addition, stigma concerns and beliefs about toughness in sport may further intensify this suppressing pathway by making athletes less willing to disclose psychological difficulties or seek professional support (Altinişik et al., 2024; Clement et al., 2015; Dagani et al., 2023; Corrigan et al., 2014). Therefore, the suppressing mediating role of athlete burnout highlights that psychological distress does not always translate into help-seeking; motivational resources and perceived psychological readiness are also necessary.

Overall, these findings indicate that psychological processes following sports injuries are not linear but rather multi-layered and dynamic. Therefore, interventions targeting athletes should not focus solely on anxiety levels but also consider burnout as a critical factor. Sport psychologists and coaches are encouraged to adopt a holistic approach that addresses not only physical recovery but also athletes' psychological wellbeing. Furthermore, enhancing athletes' knowledge about psychological help services may serve as an important intervention pathway to strengthen help-seeking attitudes. In this regard, implementing awareness-raising initiatives and psychoeducational programs within sport settings is strongly recommended.

## 5 Limitations

The findings of this study should be interpreted in light of several limitations. First, the cross-sectional design limits the ability to draw causal inferences, as the results reflect associations between variables rather than directional relationships. Although time away after injury was examined in the additional analyses, the cross-sectional design did not allow us to track how psychological responses such as burnout, injury anxiety, and help-seeking attitudes may change across different stages of injury recovery. Future longitudinal studies should examine these changes over time to provide a more robust understanding of causal and developmental mechanisms.

Second, the use of self-report measures may introduce biases such as social desirability and response bias. Future research may benefit from incorporating alternative data collection methods, such as observational or qualitative approaches.

Third, the sample consisted exclusively of individuals who had experienced sports injuries, which may limit the generalizability of the findings. Future studies including more diverse samples in terms of sport type, age groups, and levels of professionalism would enhance the generalizability of the results.

Finally, although the variables included in this study explain a substantial portion of psychological help-seeking attitudes, other potentially relevant factors—such as cultural influences, social support, and stigma—were not included in the model. Future research incorporating these variables may provide a more comprehensive understanding of help-seeking behavior.

## Data Availability

The raw data supporting the conclusions of this article will be made available by the corresponding author upon reasonable request.

## References

[B1] Akhrem A. Gazdowska Z. (2016). Analysis of the athlete burnout phenomenon: the past, the present and the future of athlete burnout research. Balt. J. Health Phys. Act. 8, 58–70. doi: 10.29359/BJHPA.08.3.07

[B2] Altinişik M. S. Sanli E. Genç A. (2024). The mediating role of social stigma anxiety related to seeking psychological help in the relationship between the impact of traumas and self-disclosure in Syrian refugees. J. Aggress. Maltreat. Trauma 33, 1418–1436. doi: 10.1080/10926771.2024.2373343

[B3] Aron C. M. Harvey S. Hainline B. Hitchcock M. E. Reardon C. L. (2019). Post-traumatic stress disorder (PTSD) and other trauma-related mental disorders in elite athletes: a narrative review. Br. J. Sports Med. 53, 779–784. doi: 10.1136/bjsports-2019-100695 31023859

[B4] Bailes J. E. Hudson V. (2001). Classification of sport-related head trauma: a spectrum of mild to severe injury. J. Athl. Train. 36, 236–243. 12937490 PMC155412

[B5] Bani-Irshid D. M. Bani-Rshaid A. M. (2022). Effect of sport injuries on the level of confidence and anxiety among athletes in different games. Int. J. Soc. Sci. Humanit. Res. 10, 139–147.

[B6] Bateman A. Morgan K. A. (2019). The postinjury psychological sequelae of high-level Jamaican athletes: exploration of a posttraumatic stress disorder–self-efficacy conceptualization. J. Sport Rehabil. 28, 144–152. doi: 10.1123/jsr.2017-0140 29091514

[B7] Brewer B. W. Chatterton H. A. (2024). Athletic identity and sport injury processes and outcomes in young athletes: a supplemental narrative review. J. Funct. Morphol. Kinesiol. 9, 1–12. doi: 10.3390/jfmk9040191 PMC11503344 39449485

[B8] Cai C. Mei Z. Yang Y. Luo S. (2025). From adversity to adaptation: the struggle between resilience and athlete burnout in stressful situations. Front. Psychol. 16, 1–12. doi: 10.3389/fpsyg.2025.1578198 PMC12162697 40519831

[B9] Caine D. Maffulli N. Caine C. (2008). Epidemiology of injury in child and adolescent sports: injury rates, risk factors, and prevention. Clin. Sports Med. 27, 19–50. doi: 10.1016/j.csm.2007.10.008 18206567

[B10] Carver C. S. Scheier M. F. Weintraub J. K. (1989). Assessing coping strategies: a theoretically based approach. J. Pers. Soc. Psychol. 56, 267–283. doi: 10.1037/0022-3514.56.2.267 2926629

[B11] Caz Ç. Kayhan R. F. ve Bardakçi S. (2019). Spor yaralanmasi kaygi ölçegi'nin türkçe'ye uyarlanmasi: geçerlik ve güvenirlik çalişmasi. Spor Hek. Derg. 54, 52–63.

[B12] Chard M. D. Lachmann S. M. (1987). Racquet sports-patterns of injury presenting to a sports injury clinic. Br. J. Sports Med. 21, 150–153. doi: 10.1136/bjsm.21.4.150 3435816 PMC1478480

[B13] Chase M. A. Magyar T. M. Drake B. M. (2005). Fear of injury in gymnastics: self-efficacy and psychological strategies to keep on tumbling. J. Sports Sci. 23, 465–475. doi: 10.1080/02640410400021427 16194995

[B14] Clement S. Schauman O. Graham T. Maggioni F. Evans-Lacko S. Bezborodovs N. . (2015). What is the impact of mental health-related stigma on help-seeking? A systematic review of quantitative and qualitative studies. Psychol. Med. 45, 11–27. doi: 10.1017/S0033291714000129 24569086

[B15] Corrigan P. W. Druss B. G. Perlick D. A. (2014). The impact of mental illness stigma on seeking and participating in mental health care. Psychol. Sci. Public Interest 15, 37–70. doi: 10.1177/1529100614531398 26171956

[B16] Covassin T. McAJIister-Deitrick J. Bleecker A. Heiden E. O. (2015). Examining time-loss and fear of re-injury in athletes. J. Sport Behav. 38, 394–403.

[B17] Dagani J. Buizza C. Ferrari C. Ghilardi A. (2023). The role of psychological distress, stigma and coping strategies on help-seeking intentions in a sample of Italian college students. BMC Psychol. 11, 1–15. doi: 10.1186/s40359-023-01171-w 37280661 PMC10243082

[B18] Dicle A. N. Ersanli K. (2015). Basa çikma tutumlarini degerlendirme ölçeginin Türkçeye uyarlama geçerlik ve güvenirlik çalismasi. Akademik Sosyal Arastirmalar Dergisi 3, 111–126.

[B19] Diehl M. Wahl H. W. Barrett A. E. Brothers A. F. Miche M. Montepare J. M. . (2014). Awareness of aging: theoretical considerations on an emerging concept. Dev. Rev. 34, 93–113. doi: 10.1016/j.dr.2014.01.001 24958998 PMC4064469

[B20] Dyrbye L. N. Leep Hunderfund A. N. Winters R. C. Moeschler S. M. Vaa Stelling B. E. Dozois E. J. . (2021). The relationship between burnout and help-seeking behaviors, concerns, and attitudes of residents. Acad. Med. 96, 701–708. doi: 10.1097/ACM.0000000000003790 33031121

[B21] Dzieciatkowska M. Horwat P. Pierudzka W. Szymanska A. Mariowska A. (2025). Depression and other mental health disorders among athletes with injuries: challenges in diagnosis, treatment, and rehabilitation. J. Educ. Health Sport 81, 1–21. doi: 10.12775/JEHS.2025.81.59996

[B22] Edwards J. L. Crisp D. A. (2017). Seeking help for psychological distress: barriers for mental health professionals. Aust. J. Psychol. 69, 218–225. doi: 10.1111/ajpy.12146

[B23] Eklund R. C. DeFreese J. D. (2015). Athlete burnout: what we know, what we could know, and how we can find out more. Int. J. Appl. Sports Sci. 27, 63–75. doi: 10.24985/ijass.2015.27.2.63

[B24] Ernst N. Eagle S. Trbovich A. Kissinger-Knox A. Bitzer H. Kontos A. P. (2022). Lower post-injury psychological resilience is associated with increased recovery time and symptom burden following sport-related concussion. Appl. Neuropsychol. Child 11, 781–788. doi: 10.1080/21622965.2021.1964966 34410842

[B25] Fischer E. H. Cohen S. L. (1972). Demographic correlates of attitude toward seeking professional psychological help. J. Consult. Clin. Psychol. 39, 70–74. doi: 10.1037/h0033152 5045289

[B26] Fischer E. H. Farina A. (1995). Attitudes toward seeking professional psychologial help: a shortened form and considerations for research. J. Coll. Stud. Dev. 36, 368–373. doi: 10.1037/t05375-000

[B27] Fischer E. H. Turner J. I. (1970). Orientations to seeking professional help: development and research utility of an attitude scale. J. Consult. Clin. Psychol. 35, 79–90. doi: 10.1037/h0029636 5487612

[B28] Foster B. J. ve Chow G. M. (2019). Development of the sport mental health continuum short form (Sport MHC-SF). J. Clin. Sport Psychol. 13, 593–608. doi: 10.1123/jcsp.2017-0057

[B29] Gomes A. R. Faria S. Vilela C. (2017). Anxiety and burnout in young athletes: the mediating role of cognitive appraisal. Scand. J. Med. Sci. Sports 27, 2116–2126. doi: 10.1111/sms.12841 28075504

[B30] Green S. L. Weinberg R. S. (2001). Relationships among athletic identity, coping skills, social support, and the psychological impact of injury in recreational participants. J. Appl. Sport Psychol. 13, 40–59. doi: 10.1080/10413200109339003

[B31] Griffiths K. M. Crisp D. A. Jorm A. F. Christensen H. (2011). Does stigma predict a belief in dealing with depression alone? J. Affect. Disord. 132, 413–417. doi: 10.1016/j.jad.2011.03.012 21440305

[B32] Gustafsson H. DeFreese J. D. Madigan D. J. (2017). Athlete burnout: review and recommendations. Curr. Opin. Psychol. 16, 109–113. doi: 10.1016/j.copsyc.2017.05.002 28813331

[B33] Hadad S. (2026). Factors influencing student help-seeking behavior during crisis: a mixed-method analysis in higher education. Stud. High. Educ. 51, 371–389. doi: 10.1080/03075079.2025.2468842

[B34] Hardy L. (1992). Psychological stress, performance, and injury in sport. Br. Med. Bull. 48, 615–629. doi: 10.1093/oxfordjournals.bmb.a072567 1450888

[B35] Haugen E. (2022). Athlete mental health & psychological impact of sport injury. Oper. Tech. Sports Med. 30, 1–10. doi: 10.1016/j.otsm.2022.150898

[B36] Hayes A. F. (2017). Introduction to Mediation, Moderation, and Conditional Process Analysis: A Regression-Based Approach. New York, NY: Guilford Publications.

[B37] Heil J. (1993). Psychology of Sport Injury. Champaign, IL: Human Kinetics Publishers.

[B38] Hoar S. D. Flint F. (2008). Determinants of help-seeking intentions in the context of athletic injury recovery. Int. J. Sport Exerc. Psychol. 6, 157–175. doi: 10.1080/1612197X.2008.9671859

[B39] Jeong L. Li D. (2024). Psychological wellbeing from sports injuries in adolescence: a narrative review. Cureus 16:64018. doi: 10.7759/cureus.64018 PMC11302481 39109136

[B40] Jones C. Gulliver A. Keegan R. (2022). A brief online video-based intervention to promote mental health help-seeking in the context of injuries for athletes: a pilot study. Psychol. Sport Exerc. 63, 1–10. doi: 10.1016/j.psychsport.2022.102281

[B41] Jones S. Agud K. McSweeney J. (2020). Barriers and facilitators to seeking mental health care among first responders: removing the darkness. J. Am. Psychiatr. Nurses. Assoc. 26, 43–54. doi: 10.1177/1078390319871997 31509058

[B42] Kaplánová A. (2024). Psychological readiness of football players for the match and its connection with self-esteem and competitive anxiety. Heliyon 10, 1–9. doi: 10.1016/j.heliyon.2024.e27608 PMC10944273 38496851

[B43] Kaygusuz S. Karagözoglu C. (2023). Sporda psikolojik toparlanma ölçegi (SPTÖ) geliştirme çalişmasi. J. Sport Sci. Res. 8, 157–174. doi: 10.25307/jssr.1192861

[B44] Kelecek S. Kara F. M. Çetinkalp F. Z. K. Aşçi F. H. (2016). Sporcu Tükenmişlik Ölçegi'nin Türkçe uyarlamasi. Spor Bil. Derg. 27, 150–161. doi: 10.17644/sbd.311371

[B45] Kenny R. Dooley B. Fitzgerald A. (2016). How psychological resources mediate and perceived social support moderates the relationship between depressive symptoms and help-seeking intentions in college students. Br. J. Guid. Counc. 44, 402–413. doi: 10.1080/03069885.2016.1190445

[B46] Keyes C. L. (2002). The mental health continuum: from languishing to flourishing in life. J. Health Soc. Behav. 43, 207–222. doi: 10.2307/3090197 12096700

[B47] Koutsimani P. Montgomery A. Georganta K. (2019). The relationship between burnout, depression, and anxiety: a systematic review and meta-analysis. Front. Psychol. 10, 1–19. doi: 10.3389/fpsyg.2019.00284 30918490 PMC6424886

[B48] Kvist J. Ek A. Sporrstedt K. Good L. (2005). Fear of re-injury: a hindrance for returning to sports after anterior cruciate ligament reconstruction. Knee Surg. Sports Traumatol. Arthrosc. 13, 393–397. doi: 10.1007/s00167-004-0591-8 15703963

[B49] Labra O. Lacasse A. Gingras-Lacroix G. Guimond F.-A. Gaboury I. Gagnon M. A. (2019). Men's help-seeking attitudes in rural communities affected by a natural disaster. Am. J. Mens Health 13, 1–12. doi: 10.1177/1557988318821512 PMC6775550 30595101

[B50] Levy A. R. Polman R. C. Clough P. J. Marchant D. C. Earle K. (2006). Mental toughness as a determinant of beliefs, pain, and adherence in sport injury rehabilitation. J. Sport Rehabil. 15, 245–254. doi: 10.1123/jsr.15.3.245

[B51] Li C. Wang C. J. Kee Y. H. (2013). Burnout and its relations with basic psychological needs and motivation among athletes: a systematic review and meta-analysis. Psychol. Sport Exerc. 14, 692–700. doi: 10.1016/j.psychsport.2013.04.009

[B52] Lien Y. J. Chen L. Cai J. Wang Y. H. Liu Y. Y. (2024). The power of knowledge: how mental health literacy can overcome barriers to seeking help. Am. J. Orthopsychiatry 94, 127–147. doi: 10.1037/ort0000708 37917500

[B53] Machado W. D. L. Bandeira D. R. (2015). Positive mental health scale: validation of the mental health continuum-short form. Psico-Usf 20, 259–274. doi: 10.1590/1413-82712015200207

[B54] Mackenzie C. S. Erickson J. Deane F. P. Wright M. (2014). Changes in attitudes toward seeking mental health services: a 40-year cross-temporal meta-analysis. Clin. Psychol. Rev. 34, 99–106. doi: 10.1016/j.cpr.2013.12.001 24486521

[B55] Mackenzie C. S. Gekoski W. L. Knox V. J. (2006). Age, gender, and the underutilization of mental health services: the influence of help-seeking attitudes. Aging Ment. Health 10, 574–582. doi: 10.1080/13607860600641200 17050086

[B56] Mackenzie C. S. Heath P. J. Vogel D. L. Chekay R. (2019). Age differences in public stigma, self-stigma, and attitudes toward seeking help: a moderated mediation model. J. Clin. Psychol. 75, 2259–2272. doi: 10.1002/jclp.22845 31385298

[B57] Malcolm D. Pullen E. (2020). Everything I enjoy doing I just couldn't do: biographical disruption for sport-related injury. Health 24, 366–383. doi: 10.1177/1363459318800142 30253661

[B58] Masten R. StraŽar K. Žilavec I. Tušak M. Kandare M. (2014). Psychological response of athletes to injury. Kinesiology. 46, 127–134.

[B59] McNally R. J. Bryant R. A. Ehlers A. (2003). Does early psychological intervention promote recovery from posttraumatic stress?. Psychol. Sci. Public Interest 4, 45–79. doi: 10.1111/1529-1006.01421 26151755

[B60] Mojtahe K. Burrichter K. (2023). The role of psychological resilience in athletic injury recovery and performance. Rev. Psicol. Deporte 32, 50–59.

[B61] Moreno X. Moreno F. (2024). Attitudes toward seeking psychological help among community dwelling older adults enrolled in primary care in Chile. BMC Geriatr. 24, 1–13. doi: 10.1186/s12877-024-04986-3 38693485 PMC11064339

[B62] Murstein B. I. Fontaine P. A. (1993). The public's knowledge about psychologists and other mental health professionals. Am. Psychol. 48, 839–845. doi: 10.1037/0003-066X.48.7.839 8357108

[B63] Nam S. K. Choi S. I. Lee J. H. Lee M. K. Kim A. R. Lee S. M. (2013). Psychological factors in college students' attitudes toward seeking professional psychological help: a meta-analysis. Prof. Psychol. Res. Pract. 44, 37–45. doi: 10.1037/a0029562

[B64] Niegocki K. L. Ægisdóttir S. (2019). College students' coping and psychological help-seeking attitudes and intentions. J. Ment. Health Couns. 41, 144–157. doi: 10.17744/mehc.41.2.04

[B65] Nippert A. H. Smith A. M. (2008). Psychologic stress related to injury and impact on sport performance. Phys. Med. Rehabil. Clin. N. Am. 19, 399–418. doi: 10.1016/j.pmr.2007.12.003 18395654

[B66] O'Brien J. Finch C. F. Pruna R. McCall A. (2019). A new model for injury prevention in team sports: the team-sport injury prevention (TIP) cycle. Sci. Med. Footb. 3, 77–80. doi: 10.1080/24733938.2018.1512752

[B67] Osborne R. E. Doty S. A. (2022). Athlete coping: personality dimensions of recovery from injury. J. Phys. Educ. 9, 1–11. doi: 10.15640/jpesm.v9a1

[B68] Padaki A. S. Noticewala M. S. Levine W. N. Ahmad C. S. Popkin M. K. Popkin C. A. (2018). Prevalence of posttraumatic stress disorder symptoms among young athletes after anterior cruciate ligament rupture. Orthop. J. Sports Med. 6:2325967118787159. doi: 10.1177/2325967118787159 30109239 PMC6083780

[B69] Panis M. P. Damayanti Y. Keraf M. K. A. (2019). Coping strategies, personality type, and help-seeking behavior for mental health problems. J. Health Behav. Sci. 1, 98–105. doi: 10.35508/jhbs.v1i2.2087

[B70] Perenc L. Radochonski M. (2016). Psychological predictors of seeking help from mental health practitioners among a large sample of polish young adults. Int. J. Environ. Res. Public Health 13:1049. doi: 10.3390/ijerph13111049 27792204 PMC5129259

[B71] Picco L. Abdin E. Chong S. A. Pang S. Shafie S. Chua B. Y. . (2016). Attitudes toward seeking professional psychological help: factor structure and socio-demographic predictors. Front Psychol. 7, 1–10. doi: 10.3389/fpsyg.2016.00547 27199794 PMC4842935

[B72] Podlog L. Eklund R. C. (2005). Return to sport after serious injury: a retrospective examination of motivation and psychological outcomes. J. Sport Rehabil. 14, 20–34. doi: 10.1123/jsr.14.1.20

[B73] Podlog L. Wadey R. Stark A. Lochbaum M. Hannon J. Newton M. (2013). An adolescent perspective on injury recovery and the return to sport. Psychol. Sport Exerc. 14, 437–446. doi: 10.1016/j.psychsport.2012.12.005

[B74] Putukian M. (2016). The psychological response to injury in student athletes: a narrative review with a focus on mental health. Br. J. Sports Med. 50, 145–148. doi: 10.1136/bjsports-2015-095586 26719498

[B75] Raedeke T. D. Smith A. L. (2001). Development and preliminary validation of an athlete burnout measure. J. Sport Exerc. Psychol. 23, 281–306. doi: 10.1123/jsep.23.4.281 28682196

[B76] Rex C. C. Metzler J. N. (2016). Development of the sport injury anxiety scale. Meas. Phys. Educ. Exerc. Sci. 20, 146–158. doi: 10.1080/1091367X.2016.1188818

[B77] Ristolainen L. Kettunen J. A. Kujala U. M. Heinonen A. (2012). Sport injuries as the main cause of sport career termination among finnish top-level athletes. Eur. J. Sport Sci. 12, 274–282. doi: 10.1080/17461391.2011.566365

[B78] Rogers D. L. Tanaka M. J. Cosgarea A. J. Ginsburg R. D. Dreher G. M. (2024). How mental health affects injury risk and outcomes in athletes. Sports Health 16, 222–229. doi: 10.1177/19417381231179678 37326145 PMC10916780

[B79] Stadden S. A. (2007). The influence of athletic identity, expectation of toughness, and attitude toward pain and injury on athletes' help-seeking tendencies [Unpublished doctoral thesis]. The University of North Carolina at Greensboro, Greensboro, NC.

[B80] Stephenson S. D. Kocan J. W. Vinod A. V. Kluczynski M. A. Bisson L. J. (2021). A comprehensive summary of systematic reviews on sports injury prevention strategies. Orthop. J. Sports Med. 9, 1–21. doi: 10.1177/23259671211035776 PMC8558815 34734094

[B81] Surgenor L. J. (1985). Attitudes toward seeking professional psychological help. NZ. J. Psychol. 14, 27–33.

[B82] Timpka T. Ekstrand J. Svanström L. (2006). From sports injury prevention to safety promotion in sports. Sports Med. 36, 733–745. doi: 10.2165/00007256-200636090-00002 16937950

[B83] Timpka T. Jacobsson J. Bickenbach J. Finch C. F. Ekberg J. Nordenfelt L. (2014). What is a sports injury?. Sports Med. 44, 423–428. doi: 10.1007/s40279-014-0143-4 24469737

[B84] Tingaz E. O. (2022). Sporcu ruh sagligi sürekliligi-kisa formu: türkçeye uyarlanmasi ve psikometrik özelliklerinin incelenmesi. Spor Bil. Derg. 33, 43–52. doi: 10.17644/sbd.948200

[B85] Topkaya N. (2010). Psikolojik yardim alma niyetinin sosyal damgalanma, tedavi korkusu, beklenen yarar, beklenen risk ve tutum faktörleriyle modellenmesi [Unpublished doctoral thesis]. Ege University, Turkey.

[B86] Topkaya N. (2014). Gender, self-stigma, and public stigma in predicting attitudes toward psychological help-seeking. Educ. Sci. Theory Pract. 14, 480–487. doi: 10.12738/estp.2014.2.1799

[B87] Turgut C. Cavkaytar S. Kocaekşi S. (2025). Evaluation of return to sport and anxiety in injured team athletes: a mixed methods study. Pamukkale J. Sport Sci. 16, 229–252. doi: 10.54141/psbd.1574316

[B88] Vann S. E. Moore M. Freiburger K. Johnson H. (2018). The end is not the injury: posttraumatic growth after sport injuries. J. Amateur Sport 4, 87–102. doi: 10.17161/jas.v4i2.6705

[B89] Verhagen E. Van Mechelen W. (Eds.). (2010). Sports Injury Research. Oxford: Oxford University Press. doi: 10.1093/acprof:oso/9780199561629.001.0001

[B90] Walker N. Thatcher J. Lavallee D. (2010). A preliminary development of the re-injury anxiety inventory (RIAI). Phys. Ther. Sport 11, 23–29. doi: 10.1016/j.ptsp.2009.09.003 20129120

[B91] Yang J. Li Y. Gao R. Chen H. Yang Z. (2023). Relationship between mental health literacy and professional psychological help-seeking attitudes in China: a chain mediation model. BMC Psychiatry 23:956. doi: 10.1186/s12888-023-05458-5 38129805 PMC10734200

[B92] Yang L. Zhang Z. Zhang J. Veloo A. (2024). The relationship between competitive anxiety and athlete burnout in college athlete: the mediating roles of competence and autonomy. BMC Psychology 12:396. doi: 10.1186/s40359-024-01888-2 39020424 PMC11256448

[B93] Yeap R. Low W. Y. (2009). Mental health knowledge, attitude and help-seeking tendency: a malaysian context. Singapore Med. J. 50, 1169–1176. 20087554

[B94] Yi S. H. Tidwell R. (2005). Adult Korean Americans: their attitudes toward seeking professional counseling services. Community Ment. Health J. 41, 571–580. doi: 10.1007/s10597-005-6362-2 16142539

[B95] Zhao R. Amanvermez Y. Pei J. Castro-Ramirez F. Rapsey C. Garcia C. . (2025). Research review: help-seeking intentions, behaviors, and barriers in college students–a systematic review and meta-analysis. J. Child Psychol. Psychiatry 66, 1593–1605. doi: 10.1111/jcpp.14145 40077833 PMC12447698

